# Can we improve the chilling tolerance of maize photosynthesis through breeding?

**DOI:** 10.1093/jxb/erac045

**Published:** 2022-02-10

**Authors:** Angela C Burnett, Johannes Kromdijk

**Affiliations:** 1 Department of Plant Sciences, University of Cambridge Cambridge, UK; 2 University of Edinburgh, UK

**Keywords:** Breeding, chilling stress, chilling tolerance, cold stress, cold tolerance, genetics, maize, photosynthesis, quantitative trait loci (QTL), spectroscopy

## Abstract

Chilling tolerance is necessary for crops to thrive in temperate regions where cold snaps and lower baseline temperatures place limits on life processes; this is particularly true for crops of tropical origin such as maize. Photosynthesis is often adversely affected by chilling stress, yet the maintenance of photosynthesis is essential for healthy growth and development, and most crucially for yield. In this review, we describe the physiological basis for enhancing chilling tolerance of photosynthesis in maize by examining nine key responses to chilling stress. We synthesize current knowledge of genetic variation for photosynthetic chilling tolerance in maize with respect to each of these traits and summarize the extent to which genetic mapping and candidate genes have been used to understand the genomic regions underpinning chilling tolerance. Finally, we provide perspectives on the future of breeding for photosynthetic chilling tolerance in maize. We advocate for holistic and high-throughput approaches to screen for chilling tolerance of photosynthesis in research and breeding programmes in order to develop resilient crops for the future.

## Teaser

Photosynthetic chilling tolerance is composed of several physiological mechanisms, underpinned by differing amounts of genetic variation. A holistic, high-throughput approach is needed to improve chilling tolerance of photosynthesis in maize.

## Introduction

Temperature is a key determinant of plant species distribution (Osmond *et al.*, 1987; Nievola *et al.*, 2017), and our planet is experiencing a rise in the frequency and severity of extreme temperature events (IPCC, 2018). At the same time, the world’s population is increasing rapidly, demanding a concomitant increase in global food production which will depend in part upon improved photosynthesis (Ort *et al.*, 2015; Simkin *et al.*, 2019). Whilst populations are stable or decreasing in many countries that grow maize, improving photosynthesis is nevertheless of relevance for maintaining crop yields in the context of temperature stresses exacerbated by climate change. In cereal crops, reproduction is the most temperature-sensitive growth stage (Yoshida *et al.*, 1981), making temperature stress a critical limitation on yield and therefore of direct relevance for food production; plant establishment and vegetative growth are also susceptible to temperature stress. Chilling temperature stress in particular is a strong limiting factor on plant growth and survival in temperate regions, where it is the primary stress impacting germination as well as affecting subsequent growth and development including crop production (Revilla *et al.*, 2005; Sanghera *et al.*, 2011). Chilling tolerance, including chilling tolerance of photosynthesis, is therefore essential if plants are to survive and even to thrive in such conditions. Improving our understanding of photosynthetic chilling tolerance in crop plants is thus both critical and timely for maintaining and increasing food production to support our growing global population.

Stress affects gene expression, metabolism, physiology, and morphology (Krasensky and Jonak, 2012). Chilling tolerance involves physiological or morphological adaptations to combat chilling stress, in contrast to chilling avoidance which is achieved by seed or vegetative dormancy (Revilla *et al.*, 2005). Chilling tolerance can occur at different time scales, which may be broadly arranged into three categories. The longest of these is adaptation to chilling stress, which occurs when plants have evolved to deal with perennially cold conditions; one example is evergreen trees down-regulating photosynthesis (Savitch *et al.*, 2002). Next, in contrast to evolutionary adaptation, acclimation to chilling stress occurs when plants are grown under cold conditions that they do not necessarily always experience; chilling-tolerant species are those which are able to acclimate to cold temperatures (Ensminger *et al.*, 2006). This acclimation involves the employment of survival strategies that are not constitutively expressed under all growth conditions, in response to a chronic chilling stress that persists for much of the season. Finally, on the shortest time scale, tolerance to acute chilling stress describes resilience to cold snaps—short periods of unexpected or unseasonal cold weather to which plants may not already be acclimated (Hüner *et al.*, 2016). This review focuses on chilling tolerance in maize (*Zea mays* L.), a species in which the chilling response has been much studied in order to facilitate the growth of this important crop in temperate regions. Maize is the most grown cereal crop in the world, making its temperature response a critical aspect of global food security. Since maize is not adapted to deal with low temperatures, we consider the responses of maize to chronic and acute chilling stresses caused by cool seasons or cold snaps, respectively.

Plant species which originated in tropical regions are often especially sensitive to chilling stress (Sanghera *et al.*, 2011), and maize is no exception (Miedema, 1982). We define chilling stress as the presence of suboptimal cool temperatures above 0 °C. Chilling and freezing temperatures impose stress in different ways: chilling stress imposes a direct temperature stress whilst freezing stress, which occurs at sub-zero temperatures, causes osmotic stress via the dehydration of cells when extracellular ice crystals are formed (Hincha and Zuther, 2020); the two stresses are both genetically and physiologically distinct (Revilla *et al.*, 2005). The specific temperatures causing chilling stress vary between species, as well as between different growth stages and different organs of the plant (Revilla *et al.*, 2005). For example, roots are especially sensitive to chilling stress, and restrictions on root growth can lead to downstream effects such as a reduced supply of water and nutrients later in development. The seed imbibition and photosynthetic initiation phases are the most chilling-sensitive phases within the seed germination and seedling growth period (Revilla *et al.*, 2005). Chilling stress can occur at temperatures ranging from 0 to 15 °C (Revilla *et al.*, 2005; Zhu *et al.*, 2007; Sanghera *et al.*, 2011; Miura and Furumoto, 2013), and temperatures within this range are used for experimental work imposing chilling stress on maize (Hu *et al.*, 2017; Frascaroli and Revilla, 2019).

Maize originated in the tropics, but has been adapted to a range of climates. European varieties of maize, such as varieties in the ‘Flint’ race, can display greater chilling tolerance than those of tropical origin. Indeed, Flint lines are often used in northern European maize breeding to provide chilling tolerance (Riva-Roveda *et al.*, 2016). In temperate regions, where maize production has been increasing for several decades (Fracheboud *et al.*, 1999; Frascaroli and Revilla, 2019), early planting increases plant biomass and reduces exposure to drought and parasites, and the associated canopy coverage decreases competition with weeds. However, early planting also increases seedling stress from chilling and disease. Overall, maize establishment is more difficult in temperate regions (Jompuk *et al.*, 2005).

Chilling stress in maize is already considered to occur at temperatures below 10–15 °C (Hu *et al.*, 2017; Frascaroli and Revilla, 2019). Generally speaking, temperatures below 15 °C slow growth, with this threshold increasing to 20 °C in more established plants, while temperatures below 5 °C cause further cell and tissue damage, and injury to seeds and seedlings (Frascaroli and Revilla, 2019). Temperatures below 10 °C badly affect maize germination (Janowiak *et al.*, 2002) and photosynthesis (Foyer *et al.*, 2002), although it should be noted that maize varieties display significant variation in chilling tolerance, as discussed below. In an agricultural setting, severe chilling stress can occur below 8 °C and maize should therefore ideally be sown when temperatures exceed this threshold (Sobkowiak *et al*., 2014, 2016; Jończyk *et al.*, 2017).

**Fig. 1.**
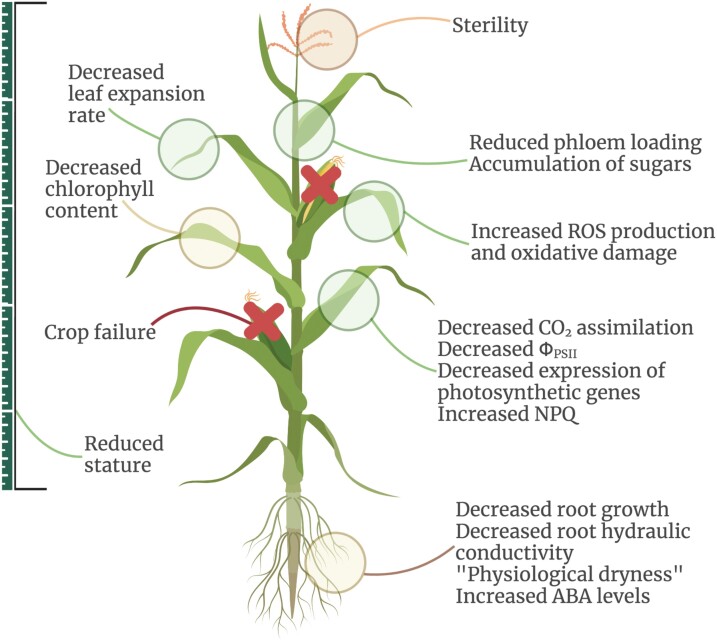
Effects of chilling stress on maize plants. The impacts of chilling temperatures on maize physiology and morphology can be observed across a range of key traits. Growth slows down or ceases entirely, which can be observed in decreased root growth, leaf expansion, and overall plant stature. The negative impact of chilling on the root system leads to decreased hydraulic conductance and partially mirrors drought stress responses, such as, for example, elevated abscisic acid (ABA) levels. Chilling also strongly impacts photosynthetic performance, which can be observed in decreases in CO_2_ assimilation, PSII operating efficiency (Φ_PSII_), and down-regulation of photosynthetic genes; this can be further compounded by the accumulation of sugars due to decreased phloem loading. In addition, photoprotection via non-photochemical quenching (NPQ) is up-regulated to mitigate the imbalance between light-dependent and independent reactions; nevertheless chilling enhances the accumulation of reactive oxygen species (ROS) as well as the breakdown of chlorophyll. Finally, chilling around the generative stages can strongly impact yield via male sterility and expansion of the anthesis–silking interval, leading to crop failure. Created with BioRender.com.

Chilling stress has multiple effects on plant morphology and function (Fig. 1). Chilling reduces plant establishment, growth, and reproduction, and leads to wilting, chlorosis, and necrosis (Revilla *et al.*, 2005). Chlorosis can be linked to cell membrane disruption (Miedema, 1982); properties of the cell wall and membrane are important for chilling tolerance (Sanghera *et al.*, 2011; Frascaroli and Revilla, 2019). Chilling stress affects metabolism, root growth and morphology, leaf area, number of days to emergence, germination rate, chlorophyll, and the efficiency of PSII, Φ_PSII_ (Janowiak *et al.*, 2002; Hund *et al.*, 2007; Frascaroli and Revilla, 2019). Cell membrane disruption, chlorophyll bleaching, and decreased Φ_PSII_ contribute to lowered rates of photosynthesis, impacting growth and productivity.

Chilling tolerance is a complex polygenic trait (Tokuhisa and Browse, 1999; Thomashow, 2001) and its genetic regulation is not well understood (Frascaroli and Revilla, 2019). Chilling tolerance in maize is regulated independently at different growth stages (Hodges *et al.*, 1997; Revilla *et al.*, 2000) and, furthermore, there are interactions between genotype and environment (Fracheboud *et al.*, 2004; Revilla *et al.*, 2005; Presterl *et al.*, 2007). Genes involved in chilling tolerance may be identified through transcriptomic, proteomic, or genomic approaches (Frascaroli and Revilla, 2019). Variation in traits of interest may be mapped to the genome using genomic markers such as single nucleotide polymorphisms (SNPs) or quantitative trait loci (QTL). SNPs are particularly useful for performing genome-wide association studies (GWAS) which can identify QTL and increase the resolution of QTL mapping (Hu *et al.*, 2017; Frascaroli and Revilla, 2019; Li *et al.*, 2021). Genetic mapping performed using SNPs can also be used for marker-assisted selection and to identify candidate genes (Miculan *et al.*, 2021; Waqas *et al.*, 2021). Once identified, candidate genes relating to physiological traits of interest may be classified according to functional characteristics using Gene Ontology (GO) terms; GO term annotations are now available for all of the protein-coding genes in maize (Wimalanathan *et al.*, 2018).

In this review, we examine the physiology of photosynthetic chilling tolerance in maize, genetic variation in photosynthetic chilling tolerance, and the genetic elements underpinning this variation, in order to address the question: Can we improve the chilling tolerance of maize photosynthesis through breeding?

## Physiology of photosynthetic chilling tolerance

In order to survive a period of chilling stress, plants must adjust their physiological processes to minimize damage. Maize plants display a range of chilling responses (Fig. 1), and these occur on different time scales. In this section, we examine the major physiological responses to chilling stress in maize, including both immediate and longer term responses, which enable plants to react to acute and chronic chilling stress. Some responses are indicative of protective mechanisms that mitigate the effects of chilling stress, whilst other responses reveal that damage has already occurred. We outline three categories of physiological response to chilling stress, organized according to the time scales in which they have been reported to occur: photosynthetic responses; photoprotective responses; and signalling and developmental responses. Finally, to conclude this section, we consider the most appropriate physiological measurements for screening natural genetic variation in photosynthetic chilling tolerance.

### Photosynthetic responses to chilling: carbon assimilation

Maize carries out C_4_ photosynthesis, which involves a biochemical carbon-concentrating mechanism that helps to increase photosynthetic efficiency, especially under hot and dry conditions. Atmospheric CO_2_ equilibrates with bicarbonate and is firstly fixed—via phosphoenolpyruvate carboxylase (PEPC; BRENDA: EC 4.1.1.31)—into the 4-carbon metabolite oxalo-acetate in the mesophyll. Oxalo-acetate is converted to malate which diffuses along a concentration gradient inwards from the mesophyll to the bundle sheath cells. In the chloroplasts of the bundle sheath cells where Rubisco (BRENDA: EC 4.1.1.39) is compartmentalized, decarboxylation of malate mediated by NADP-malic enzyme (BRENDA: EC 1.1.1.40) releases CO_2_ while reducing NADP^+^ to NADPH. This carbon-concentrating mechanism augments the CO_2_:O_2_ ratio and thus increases the efficacy of ribulose bisphospate (RuBP) carboxylation by Rubisco in the Calvin–Benson–Bassham cycle by competitive inhibition of RuBP oxygenation.

**Fig. 2.**
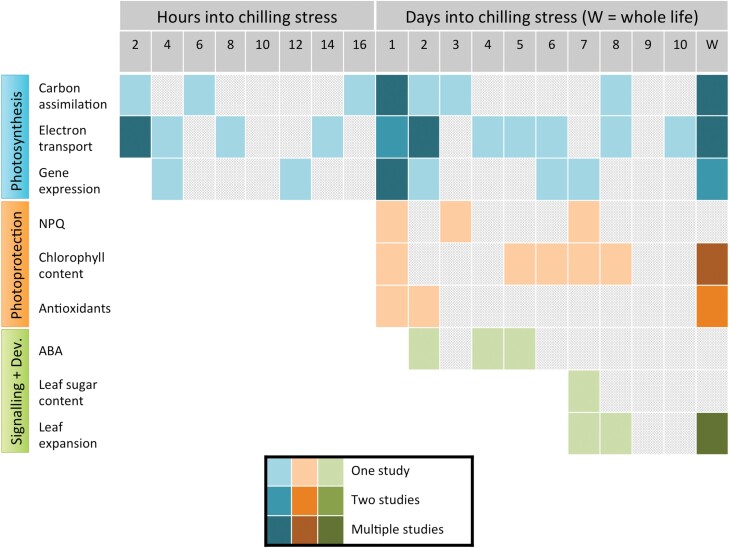
Timeline of maize responses to chilling stress for nine physiological variables. Variables are grouped in three categories: photosynthetic responses in blue, photoprotective responses in orange, and signalling and developmental responses in green. Grey hatching indicates the projected time range during which the response is expected to occur, with confirmed time points indicated by coloured boxes. The darker the colour, the greater the number of studies reviewed here that reported the trend at any given time point. Studies included here do not necessarily include genetic variation, but must demonstrate the relevant response to chilling stress in at least one maize line. Many studies reveal effects following a treatment lasting the duration of the experiment, denoted by ‘W’ for the whole experimental life-span. NPQ, non-photochemical quenching; ABA, abscisic acid.

The initial physiological responses to chilling stress in maize are related to carbon assimilation. Firstly, the capacity and rate of net CO_2_ assimilation decrease (Fig. 2) when plants are temporarily exposed to chilling stress. This can already be observed after 2 h exposure to 4 °C chilling stress and was more pronounced after a longer chilling stress of 16 h (Ying *et al.*, 2002), as well as being observed after a chilling stress of 6 h (Aguilera *et al.*, 1999). In both of these studies, measurements of CO_2_ assimilation were made during a recovery period following the chilling stress period. The decrease in net CO_2_ assimilation rate is a highly sustained response, which has been reported in many studies after 1 d (Dwyer and Tollenaar, 1989; Ying *et al.*, 2000; Aroca *et al.*, 2001; Riva-Roveda *et al.*, 2016), 2–3 d (Ying *et al.*, 2000), and 8 d of chilling stress (Lee *et al.*, 2002). The measurements by Dwyer and Tollenaar and those by Ying *et al.* were carried out during recovery after chilling stress, whilst the other studies performed measurements during the chilling stress treatment, indicating that measurements both during and after a chilling stress period may be used to measure decreased CO_2_ assimilation that occurs during chilling and persists during recovery. A decrease in CO_2_ assimilation rate was also reported in several studies which imposed a chilling stress for the duration of the experimental period (Nie and Baker, 1991; Kingston-Smith *et al.*, 1999; Ying *et al.*, 2002; Fracheboud *et al.*, 2004; Zaidi *et al.*, 2010; Rodríguez *et al.*, 2014).

Various mechanisms may contribute to the sustained decrease in CO_2_ assimilation including reduced enzyme activity, collapse of metabolic gradients between mesophyll and bundle sheath cells, damage to the photosystems, and increased diffusive limitations to CO_2_ uptake. Photosynthetic enzyme activities are often reduced under chilling stress (Avila *et al.*, 2018). The activities of fructose bisphosphatase (BRENDA: EC 3.1.3.11), Rubisco, and PEPC decrease in chilled maize leaves (Kingston-Smith *et al.*, 1997). At cooler temperatures, the speed of atomic movement and the rate of collisions decrease; many enzymatic processes are attuned to operate best within a range of optimal temperatures and will therefore perform relatively poorly outside of the relevant range. Rubisco has been speculated to be especially limiting in chilling conditions in C_4_ species, since C_4_ plants contain less Rubisco and because Rubisco is operating closer to its maximum capacity due to the high concentration of CO_2_ created by the carbon-concentrating mechanism (Sage and McKown, 2006). Furthermore, enhanced degradation of photosynthetic gene products under chilling stress reduces the amounts of enzymes in the leaf: protein breakdown is increased at low temperatures (reviewed by Sales *et al.*, 2021). Specifically, the photosynthetic enzymes pyruvate, phosphate dikinase (PPDK; BRENDA: EC 2.7.9.1), PEPC, and Rubisco break down more easily under chilling conditions in C_4_ species (Kingston-Smith *et al.*, 1997; Du *et al.*, 1999; Chinthapalli *et al.*, 2003). This increased lability means that greater enzyme synthesis is required to maintain a given activity, and therefore decreases the overall enzyme activity across the leaf. The amounts and activities of enzymes can also trade off against one another as part of the chilling stress response. For example, a decrease in Rubisco content coupled with an observed increase in Rubisco activation state may indicate an up-regulation of activation in order to compensate for the lower enzyme content observed during a chronic chilling stress experiment in maize (Kingston-Smith *et al.*, 1999).

While stomatal conductance is usually not a strong constraint to photosynthesis in maize, it also decreases strongly under chilling conditions (Lee *et al.*, 2002), which may enhance the diffusional limitation to CO_2_ uptake. However, since CO_2_ assimilation and stomatal conductance are strongly coordinated, the stomatal closure response is more likely to be a reflection of the chilling-induced decreases in CO_2_ assimilation.

### Photosynthetic responses to chilling: electron transport

As well as a decrease in net CO_2_ assimilation, chilling stress causes a decrease in the operating efficiency of PSII in the light (Φ_PSII_), which is derived from measurements of chlorophyll fluorescence (Maxwell and Johnson, 2000; Baker, 2008). Down-regulation of Φ_PSII_ in response to chilling occurs in parallel with changes in CO_2_ assimilation, being observed as early as 2 h into chilling stress (Fig. 2), measured both directly during chilling stress (Fracheboud *et al.*, 2002) and via a decrease in the maximum quantum efficiency of PSII photochemistry, *F*_v_/*F*_m_, determined during recovery following 2 h of chilling (Ying *et al.*, 2002). Decreases in *F*_v_/*F*_m_ were also reported at 2, 4, and 8 h into a chilling stress period, with greater decreases observed as time progressed (Dolstra *et al.*, 1994). The down-regulation of Φ_PSII_ has also been reported a few hours after the imposition of chilling stress (Sobkowiak *et al.*, 2014); after 1 d of chilling stress (Sobkowiak *et al*., 2014, 2016), and after 2 d (Janowiak *et al.*, 2002; Sobkowiak *et al.*, 2014; Urrutia *et al.*, 2021), 4 d (Urrutia *et al.*, 2021), 5 d (Sobkowiak *et al.*, 2014), 6 d (Urrutia *et al.*, 2021), 8 d (Lee *et al.*, 2002), and 10 d (Kościelniak *et al.*, 2005). Each of these results was obtained during the period of chilling stress, although the study by Janowiak *et al.* also included measurements made during a recovery period which are not reported here. In the case of the measurements by Kościelniak *et al.*, the chilling stress was even augmented at the time of measurement, with measurements made at 6 °C following a period of 10 d at 15 °C. As has been demonstrated for the CO_2_ assimilation rate, the chilling-induced decrease in Φ_PSII_ persists during prolonged periods of chilling stress, being reported by studies imposing chilling stress for the duration of the experiment (Fracheboud *et al.*, 1999; Kingston-Smith *et al.*, 1999, 2004; Hund *et al.*, 2007).

Balancing Φ_PSII_ with CO_2_ assimilation enables plants to maintain an appropriate energy balance, regulated by redox and pH changes as well as calcium signalling initiated by changes in plasma membrane fluidity (Ensminger *et al.*, 2006). CO_2_ assimilation and Φ_PSII_ are correlated, but the relationship between them is not always constant. For example, the relationship between CO_2_ assimilation and Φ_PSII_ can change under chilling conditions, with higher values of Φ_PSII_ relative to CO_2_ assimilation (Fryer *et al.*, 1998). However, this is not always the case, with other studies reporting a more sustained relationship between CO_2_ assimilation and Φ_PSII_ during chilling stress (Kingston-Smith *et al.*, 1997; Foyer *et al.*, 2002), particularly when irradiance is stable (Earl and Tollenaar, 1998). PSII is chronologically the first of two photosystems in linear photosynthetic electron transport, which produces ATP and reductant (NADPH) for subsequent use in the C_4_ acid shuttle and the Calvin–Benson–Bassham cycle to assimilate CO_2_ into carbohydrates. PSII is typically thought to be more susceptible to chilling stress than PSI (Kočová *et al.*, 2009). The PSII reaction centre protein D1 is easily damaged, leading to photoinhibition and reduced rates of photosynthesis; this can occur in chilling conditions, particularly when irradiance is high (Farage and Long, 1987). However, PSI is also easily damaged under chilling conditions and sharp fluctuations in light intensity (Kono *et al.*, 2014).

Down-regulation of photosynthetic electron transport may not just be a result of run-away damage to the photosystems under chilling conditions. Instead, reversible down-regulation of PSII activity via non-photochemical quenching (NPQ), or more long term via halting the D1 protein repair cycle, may also be initiated under chilling conditions in order to balance carbon sources and sinks and to reduce the potentially damaging effects of excessive light energy and concomitant production of reactive oxygen species (ROS; Ensminger *et al.*, 2006). Chilling stress reduces the metabolic sink, and photosynthesis must respond in order to maintain an appropriate carbon source:sink balance which is essential for maintaining healthy growth (Ensminger *et al.*, 2006; Burnett *et al.*, 2016; White *et al.*, 2016). Evidence for this hypothesis comes from other plant species including evergreens and *Arabidopsis*, in which chilling acclimation led to alterations to the thylakoid membrane, sucrose synthesis enzyme expression, and Calvin cycle enzyme expression, all of which have been identified as balancing regulators of the carbon source and sink enabling photosynthetic acclimation to chilling stress (Hüner *et al*., 1998, 2003; Stitt and Hurry, 2002). In maize, expression of a sucrose synthase increased in response to chilling stress (Urrutia *et al.*, 2021), as has been seen in *Arabidopsis* (Stitt and Hurry, 2002), and down-regulation of the expression of photosynthetic enzymes is also observed, as we outline in the following section.

### Photosynthetic responses to chilling: gene expression

Following soon after the down-regulation of CO_2_ assimilation and Φ_PSII_ is a down-regulation of photosynthetic gene expression (Fig. 2). While not as rapid as the decreases in CO_2_ assimilation and PSII operating efficiency, down-regulation of photosynthetic gene expression (i.e. the abundance of photosynthesis-related transcripts) has been reported as early as 4 h after the beginning of chilling stress (Li *et al.*, 2019), after 12 h in another study (Yu *et al.*, 2021), and after 1 d of chilling stress in several studies (Trzcinska-Danielewicz *et al.*, 2009; Zhang *et al.*, 2009; Jończyk *et al.*, 2017; Avila *et al.*, 2018; Banović Đeri *et al.*, 2021). A decrease in photosynthetic protein accumulation occurs soon after, reported after 2 d of chilling stress (Urrutia *et al.*, 2021). This decrease may be caused by the transcriptional or translational down-regulation of photosynthetic genes leading to a reduction in protein synthesis; by the damage and breakdown of photosynthetic proteins discussed above; by damage to the cellular machinery responsible for the synthesis and repair of proteins; or by a combination of these factors. Similarly to the other photosynthetic responses detailed in this section, the down-regulation of the expression of genes involved in photosynthesis persists during longer periods of chilling stress, being reported after 6 d (Szalai *et al.*, 2018), 7 d (Riva-Roveda *et al.*, 2016), and in long-term studies of chilling stress (Nie and Baker, 1991; Kingston-Smith *et al.*, 1999). This sustained response of down-regulation of gene expression contributes to the sustained low rates of photosynthesis observed over long periods of chilling stress.

### Photoprotective responses to chilling: NPQ, chlorophyll content, and reactive oxygen species

Since enzymatic reactions are more strongly affected than the photochemical electron transfer processes on the thylakoid membrane, chilling can lead to over-reduction of electron transfer components, and can promote production of damaging ROS. As a result, exposure to chilling tends to induce photoprotective responses to mitigate these issues. Three potentially photoprotective responses can already be seen after 1 d of chilling stress in chilling-tolerant maize plants: increased levels of NPQ, a decrease in chlorophyll content, and an alteration in antioxidant enzymes or oxidative damage (Fig. 2). These responses should be interpreted with caution, as each of these potentially photoprotective mechanisms may also be a reflection of damage caused by chilling stress.

NPQ is a compound term that encompasses a range of different non-photochemical quenching mechanisms to dissipate excitation energy in the light-harvesting antennae (reviewed by Malnoë, 2018). Some forms of NPQ are readily reversible such as energy-dependent quenching (*q*_E_), which is primarily controlled by the pH of the thylakoid lumen. In contrast, photoinactivation of the PSII reaction centre protein D1 gives rise to a sustained photoinhibitory *q*_I_-type quenching, namely quenching which leads to a long-term depression of the quantum yield of CO_2_ fixation. Unlike *q*_E_ which may be activated or deactivated within minutes, *q*_I_ is not rapidly reversible as it requires molecular repair. A decrease in *F*_v_/*F*_m_ after dark-acclimation indicates the presence of photoinhibition (Fracheboud *et al.*, 1999). Increases in NPQ have been observed after 1 d of chilling stress in some maize lines (Fig. 2), and may continue after an additional 2 d or 6 d depending on the line (Riva-Roveda *et al.*, 2016; measurements made during chilling stress). Further resolving the different forms of NPQ that are specifically up-regulated in response to chilling will be important for elucidating the photoprotective or photoinhibitory nature of these responses.

A strong decrease in leaf chlorophyll content can often be observed in young maize plants grown under suboptimal temperature. This phenotype may be a manifestation of excessive oxidative damage to chlorophylls in the light-harvesting antennae leading to photobleaching, but may also form part of a reorganization and restructuring of the light-harvesting capacity as a photoprotective response to chilling stress (Ensminger *et al.*, 2006). A decrease in chlorophyll content can already be observed after 1 d of chilling stress (Avila *et al.*, 2018). This effect has also been reported after 5 d (Aroca *et al.*, 2001), 6 d (Szalai *et al.*, 2018), 7 d (Riva-Roveda *et al.*, 2016), and after 8 d (Lee *et al.*, 2002; Fig. 2). These measurements were all performed during the chilling stress period, although Aroca *et al.* (2001) also included a subsequent recovery period, not reported here. Furthermore, a decrease in chlorophyll content is a highly sustained response to chilling stress, with multiple studies reporting decreased chlorophyll content after a chilling stress that was imposed for the whole life of the maize plants prior to measurement, suggesting that the potential for acclimation may be limited (Nie and Baker, 1991; Kingston-Smith *et al.*, 1999; Fracheboud *et al.*, 2004; Hund *et al.*, 2007; Rodríguez *et al.*, 2008).

Closely intertwined with chilling effects on leaf chlorophyll content, alterations in antioxidant capacity also manifest after 1 d of chilling stress (Fig. 2). Increased antioxidant enzyme activities were found in a chilling-tolerant maize variety (Aroca *et al.*, 2001), whereas the antioxidant molecule ascorbic acid decreased after 30 h of chilling stress in chilling-sensitive sweet-corn seedlings (Xiang *et al.*, 2020), both measured during chilling stress. Alterations to antioxidant capacity can be very persistent in response to long-term chilling. Increases in several antioxidant enzyme activities were observed across a range of maize genotypes in response to 26 d of chilling stress. In this case, superoxide dismutase, ascorbate peroxidase, and glutathione reductase all showed increased activity, whilst the response of catalase activity was dependent on the genotype (Kočová *et al.*, 2009). These changes in anti-oxidant capacity may impact accumulation of ROS. For example, increased hydrogen peroxide levels were observed in leaves exposed to 14 °C (Kingston-Smith *et al.*, 1999), which may reflect enhanced oxidative stress levels. In maize, the localization patterns of antioxidant enzymes between bundle sheath and mesophyll tissue (Doulis *et al.*, 1997) increase the propensity for oxidative damage (Kingston-Smith *et al.*, 1999; Foyer *et al.*, 2002). Reduced metabolite transport between the bundle sheath and mesophyll tissues under chilling conditions increases oxidative stress in the bundle sheath, since antioxidant enzymes are primarily localized in the mesophyll (Kingston-Smith and Foyer, 2000).

### Signalling and developmental responses to chilling: ABA, leaf sugar content, and leaf expansion

Lastly, we outline three responses related to signalling and development that occur in response to chilling stress (Fig. 2). The fastest of these three is an increase in the level of abscisic acid (ABA) which was already observable after 2 d as well as after 4 d and 5 d of chilling stress, and correlates with chilling tolerance (Capell and Dörffling, 1993; Janowiak and Dörffling, 1996; Janowiak *et al.*, 2002), and has also been confirmed under field conditions (Janowiak *et al.*, 2003). It is well known that ABA is involved in the response to drought stress and exhibits crosstalk with several metabolic and regulatory pathways (Ensminger *et al.*, 2006; Sreenivasulu *et al.*, 2012; Munemasa *et al.*, 2015; Sah *et al.*, 2016; Zhu, 2016). Guard cells are subject to ABA regulation, which stimulates stomatal closure during drought. In chilling stress conditions, ABA may therefore contribute to a sustained decrease in stomatal conductance to CO_2_ such as has been reported by Lee *et al.* (2002).

While increased ABA levels can occur relatively rapidly, a longer term response to chilling stress can be seen in the leaf soluble sugar content, which has been reported to increase after 7 d of chilling, measured during the chilling stress period (Riva-Roveda *et al.*, 2016). This increase could be a result of a decrease in phloem loading, due to chilling-induced restrictions on transport (Krapp and Stitt, 1995; Ainsworth and Bush, 2011). Alternatively, the increase in foliar sugar content may be a physiological response to maintain turgor pressure when water transfer from the roots is impaired by chilling stress. The accumulation of foliar sugars initiates negative feedback repression of photosynthesis (Krapp and Stitt, 1995; Smeekens *et al.*, 2010), which may contribute to the sustained reduction in net CO_2_ assimilation discussed above.

Finally, long-term exposure to chilling leads to a pronounced reduction in growth rate, which can be observed very clearly in a decline of leaf expansion rate. This common phenotype is often included in studies examining plants over multiple days of chilling (e.g. Riva-Roveda *et al.*, 2016). The slowing of leaf expansion and appearance rate can be striking. For example, the time taken to reach the leaf 8 stage (the growth stage at which leaf 8 is the most recent fully expanded leaf, where leaf 8 is the eighth leaf to appear on the plant) was tripled after 8 d of chilling stress at 15/13 °C at the leaf 7 stage compared with plants grown under control conditions (Lee *et al.*, 2002). To account for the decreased rate of leaf expansion under long-term chilling conditions, many studies compare control and chilling-treated plants at the same developmental stage rather than at the same time point (Fracheboud et al., 1999, 2002, 2004). However, this can give rise to extreme age differences between treatment and control groups, where the chilling-treated plants can sometimes take twice as long to reach the same developmental stage (Rodríguez *et al.*, 2008). Whilst increases in foliar ABA and soluble sugars have not yet been demonstrated to be sustained effects, a decrease in leaf expansion rate is clearly a persistent effect during chilling stress.

### Screening for chilling stress responses

Considering the nine responses outlined in this section (Fig. 2), the four most studied components of the physiological response to chilling stress are consistent between studies focused on exploring effects of chilling on physiological processes and studies focused on examining genetic variation in chilling tolerance. These four components are the three ‘photosynthesis’ parameters, and chlorophyll content. However, the degree to which each parameter is used varies between physiology- and genetics-focused studies. Assessing the studies of the photosynthetic chilling response in maize reviewed here, in physiological studies net CO_2_ assimilation rate is the most frequently studied parameter, followed by photosynthetic gene expression, Φ_PSII_, and chlorophyll content. In contrast, in genetics-focused studies, this order is reversed, with chlorophyll content being the most frequently studied parameter, followed by Φ_PSII_, photosynthetic gene expression, and net CO_2_ assimilation. In both types of study, the remaining five responses (NPQ, antioxidant enzymes or antioxidant damage, ABA, leaf sugar content, and leaf expansion) are used relatively less frequently.

The different emphasis on each of the three photosynthesis parameters and chlorophyll content between physiology- and genetics-focused studies reflect the different priorities of the two types of study. For studies of genetic variation, chlorophyll content and Φ_PSII_ provide rapid, relatively high-throughput proxies for chilling stress which are useful for screening large populations and carrying out genetic mapping, whilst measurements of the net CO_2_ assimilation rate are less high-throughput but provide more physiological detail and are therefore favoured by studies focusing on the physiological responses of maize to chilling stress. Regarding the proxies for photosynthetic chilling tolerance favoured by genetics-focused studies, we note that chlorophyll fluorescence is a particularly valuable screening tool (Fracheboud *et al.*, 1999; Baker, 2008). Specifically, Φ_PSII_ provides a useful means of distinguishing between chilling-tolerant and chilling-susceptible lines, and has been used in initial breeding attempts to enhance chilling tolerance (Fracheboud *et al.*, 1999). Fluorescence measurements are non-destructive, facilitating repeated measurements during an experimental time course. Chlorophyll content may be measured destructively using pigment analysis following extraction in solvent, but may also be estimated non-destructively from transmittance at a few specific wavelengths with a SPAD meter or more elaborate spectrometry (Avila *et al.*, 2018), both providing great rapidity and the ability to repeat measurements throughout a time course compared with biochemical pigment analysis. A major advantage of chlorophyll fluorescence over chlorophyll content is the versatility and available diversity of fluorescence measurements. Depending on the instrument and protocol used, a measurement of a few minutes may suffice to provide *F*_v_/*F*_m_, Φ_PSII_, and NPQ.

However, since both Φ_PSII_ and chlorophyll content may be decreased during stress for protective reasons or due to photodamage, it is advantageous to include a metabolic component such as net CO_2_ assimilation rate or leaf sugar content in parallel to allow more robust conclusions about the nature of the chilling response to be drawn. The time scale of the response is also relevant: short-term down-regulation of Φ_PSII_ or initiation of NPQ could be a photoprotective response, whilst long-term differences in Φ_PSII_ between genotypes are more likely to indicate variation in the capacity for sustained photosynthesis under chilling conditions and may therefore reveal chilling tolerance or susceptibility.

## Genetic variation in chilling tolerance

Having established the primary physiological responses to chilling stress in maize, we now examine the evidence for genetic variation within maize germplasm across these responses. Our focus is on naturally occurring genetic variation, which provides a useful pool of resources for breeding plants with greater chilling tolerance of photosynthesis (Faralli and Lawson, 2020). Evidence for genetic variation in photosynthetic chilling tolerance can become apparent whenever lines with contrasting chilling tolerance are studied. Studies containing two—or a few—lines may be used to identify differentially expressed genes (DEGs) in response to chilling between tolerant and susceptible lines. In contrast, large populations with sufficient phenotypic variation and tractable genotypic variation are needed for the identification of QTL or SNPs that significantly correlate with variation in chilling tolerance.

Reflecting on the nine physiological responses identified in the previous section, several studies have looked at gene expression changes in conjunction with chilling treatments in tolerant and susceptible maize lines, and candidate genes have been identified for chilling-related variation in CO_2_ assimilation rate, Φ_PSII_, photosynthetic gene expression, chlorophyll content, antioxidant capacity, leaf sugar content, and morphology related to leaf expansion (summarized in Table 1), but not for NPQ or ABA. In addition, several studies have used chilling-related variation in CO_2_ assimilation rate, Φ_PSII_, photosynthetic gene expression, NPQ, and chlorophyll across mapping populations to identify QTL for each of these traits. SNPs significantly correlated with variation in CO_2_ assimilation, Φ_PSII_, chlorophyll, and morphology related to leaf expansion have also been identified (Table 1). In contrast, we could not find any studies where genetic mapping was used for variation in antioxidant capacity, ROS accumulation and oxidative damage, ABA, or leaf sugar content in response to chilling (Table 1). Genetics-focused studies of photosynthetic chilling tolerance typically measure CO_2_ assimilation, Φ_PSII_, photosynthetic gene expression, and chlorophyll content. Considering these four traits, some general trends emerge in studies that have examined genetic variation in two or more genotypes (Table 1). Overall, decreases in CO_2_ assimilation, Φ_PSII_, and chlorophyll content are generally less pronounced in chilling-tolerant genotypes compared with chilling-sensitive genotypes, indicating that lower values of Φ_PSII_ and chlorophyll content may be more likely to reflect the result of photodamage rather than photoprotection in chilling-sensitive lines.

**Table 1.** Genetic mapping and candidate genes for nine physiological responses to chilling stress in maize

Study	Genetic variation	Genetic mapping	Candidate genes
**CO**_**2**_ **assimilation rate**			
F_2:3_ population from chilling-tolerant (ETH-DH7) and chilling-sensitive (ETH-DL3) lines. 15/13 °C for whole life following establishment; measured leaf 3 (Fracheboud *et al.*, 2004)	Yes	Yes—QTL for carbon exchange rate (a measurement of CO_2_ assimilation)^*a*^	No
233 RILs from drought-tolerant (Ac7643) and drought-susceptible (Ac7729/TZSRW) lines. 15/13 °C for whole life following establishment; measured leaf 3 (Fracheboud *et al.*, 2002)	Yes	Yes—QTL for CO_2_ fixation; eight regions with QTL for photosynthetic traits; pericentromeric region of chromosome 3 a key location^*b*^	No
226 F_2:3_ families from ETH-DH7×ETH-DL3 and 168 F_2:4_ from Lo964×Lo1016 (different chilling tolerance at germination and different root morphology). 15/13 °C for 14 d following establishment; measured leaf 3 (Hund *et al.*, 2005)	Yes	Yes—QTL for carbon exchange rate	No
282 inbred lines. 8 °C at germination (Hu *et al.*, 2017)	Carbon exchange rate not measured directly	Yes—SNPs related to carbon exchange rate in other studies	Yes—identified 18 candidate genes in total^*c*^
49 inbred lines. 15/13 °C at 7 leaf stage, measured leaf 8 (Lee *et al.*, 2002)	Yes	No	No
**PSII operating efficiency (**Φ_**PSII**_)			
F_2:3_ population from ETH-DH7×ETH-DL3. Early and late sowing in the field provided chilling treatment (Jompuk *et al.*, 2005)	Yes	Yes—QTL for Φ_PSII_ located on chromosomes 2, 4, 6, 8, 9 (most prominent on 6)	No
Population from chilling sensitive×tolerant inbred lines. 14/8 °C for the duration of the experiment. (Rodríguez *et al.*, 2014)	Yes	Yes—two QTL for maintenance of Φ_PSII_ in chilling stress^*d*^	No
Fracheboud *et al.* (2004)	Yes	Yes—QTL for Φ_PSII_	No
Fracheboud *et al.* (2002)	Yes	Yes—QTL for Φ_PSII_	No
Hund *et al.* (2005)	Yes	Yes—QTL for Φ_PSII_, located on different chromosomes in the different populations	No
168 F_2:4_ families from Lo964×Lo1016 (see above). 15/13 °C for the duration of the experiment; measured at first leaf stage (Hund *et al.*, 2004)	Yes	Yes—four QTL for Φ_PSII_	A locus for Φ_PSII_ was identified
One chilling-tolerant and one chilling-sensitive line. (ETH-DH7 and ETH-DL3). 8/6 °C imposed for 14 h at third leaf stage (Sobkowiak *et al.*, 2014)	Yes	Yes—DEGs adjacent to QTL for chlorophyll fluorescence	Yes—overall, identified 66 genes responding differently between lines (DEGs)
Lee *et al.* (2002)	Yes	No	No
Two panels: 306 Dent lines and 292 Flint lines. 14/8 °C for duration of experiment (Revilla *et al.*, 2016)	Yes	Yes—two SNPs for Φ_PSII_ in chilling stress in Flint population (chromosomes 1, 4); QTL for Φ_PSII_. Overall, more QTL for chilling tolerance were identified in the Flint panel	Yes—performed GWAS and identified candidate genes
Three breeding groups, total 375 inbred lines. 16/13 °C. (Strigens *et al.*, 2013)	Yes—significant differences in ϕ_PSII_ between the breeding groups	Yes—identified three QTL for Φ_PSII_ (two under chilling stress, one only under optimal conditions)	No
**Photosynthetic gene expression**			
Sobkowiak *et al.* (2014)	Yes	Yes—DEGs adjacent to QTL for C_4_ enzymes	Yes (see above)
One chilling-tolerant (S68911) and two chilling-sensitive lines (S160 and S50676). 14/12 °C for 4 d then 8/6 °C for 4 d at third leaf stage (Sobkowiak *et al.*, 2016)	Yes	No	Yes—GO enrichment identified photosynthetic genes
Two unrelated inbred lines: CG60, CG102. 14/2 °C for 3 d at second leaf stage; measured after 1 d chilling (Avila *et al.*, 2018)	Yes	No	Yes—GO term analysis identified photosynthetic genes down-regulated in chilling stress
Four stress-sensitive ‘Lancaster’ lines, four tolerant lines. 6/4 °C for 24 h at fourth leaf stage (Banović Đeri *et al.*, 2021)	Yes	No	Yes—seven DEGs including photosynthetic genes. Differential expression between genotypes and treatment/control and between genotypes
One chilling-tolerant (M54), one chilling-sensitive (753F) line. 4 °C chilling stress for up to 24 h at fourth leaf stage (Li *et al.*, 2019)	Yes	No	Yes—chilling stress affected photosynthetic genes
One chilling-tolerant (B144), one-chilling sensitive (Q319) line. 5 °C chilling stress for 12 h or 24 h at third leaf stage (Yu *et al.*, 2021)	Yes	No	Yes—up-regulation of the D1 protein *psb29* after 24 h (following initial decrease at 12 h) enabled B144 to protect PSII from photooxidation
**Non-photochemical quenching (NPQ)**			
Fracheboud *et al.* (2002)	Yes	Yes—QTL for xanthophylls	No
A chilling-sensitive inbred line (A661) and B73. 15 °C for the duration of the experiment (Rodríguez *et al.*, 2013)	Yes—lower xanthophylls in A661	No	No
**Chlorophyll content**			
302 RILs from B73×Mo17. 14/8 °C for the experiment duration; measured after 30 d (Rodríguez *et al.*, 2008)	Yes—measured chlorophyll using optical scale	Yes—QTL identified on chromosomes 3 and 6, under chilling conditions only^*e*^	QTL on chromosome 6 may correspond to *luteus11* locus
Fracheboud *et al.* (2004)	Yes	QTL identified on chromosome 3	No
Fracheboud *et al.* (2002)	Yes	Yes—QTL for chlorophyll	No
Hund *et al.* (2005)	Yes	Yes—QTL for chlorophyll	No
Hu *et al.* (2017)	Chlorophyll not measured directly	Yes—SNPs related to chlorophyll in other studies	Yes—see above
Two populations of field×sweet corn (B73×P39, 179 RILs; B73×IL14 h, 213 RILs). 14/10 °C for the experiment duration (Allam *et al.*, 2016)	Yes	Yes—QTL for chlorophyll content	No
Hund *et al.* (2004)	Yes	Yes—seven QTL for chlorophyll	No
76 accessions. 10/8 °C for whole life, measured at fourth leaf stage (Bano *et al.*, 2015)	Yes	No	No
Sobkowiak *et al.* (2014)	Chlorophyll not measured directly	Yes—DEGs adjacent to QTL for chlorophyll content	Yes—see above
Lee *et al.* (2002)	Yes	No	No
Jompuk *et al.* (2005)	Yes	Yes—six QTL on chromosomes 1, 2, 3, 4, 10 in early-sown plants; four QTL in late-sown plants^*f*^	No
Avila *et al.* (2018)	Yes	No	Differential expression of chloroplast genes under chilling stress
Rodríguez *et al.* (2013)	Yes—lower chlorophyll and higher chlorophyllase activity in A661	Yes—QTL on chromosome 2 for chilling-induced albinism^*g*^	Yes—a putative gene in chlorophyll biosynthesis, and a chlorophyll-binding protein
Revilla *et al.* (2016)	Yes	Yes—two SNPs for chlorophyll in chilling stress in Dent population (chromosomes 1, 4)	Yes
**Antioxidant enzymes, or oxidative damage**			
Association panel of 125 inbred lines. 6.4 °C for 7 d at third leaf stage (Huang *et al.*, 2013)	Not measured directly	No	Candidate genes in five categories including one for antifreeze and H_2_O_2_ removal
Sobkowiak *et al.* (2014)	Not measured directly	Yes—DEGs adjacent to QTL related to antioxidant levels	Genes for antioxidant systems identified
Tolerant (S68911) and sensitive (B73) inbred lines. 14/10 °C for the duration of the experiment, measured at early growth stages (Jończyket al., 2021)	Not measured directly, but transcriptomic data suggest greater ROS scavenging in S68911 in chilling conditions	No	No; examined stress-response motifs and chromatin accessibility, related to chilling tolerance in the tolerant line which switched from growth to defence
**Abscisic acid (ABA)**			
Jończyk *et al.* (2021)	Not measured directly, but transcriptomic data suggest greater ABA synthesis in tolerant line in chilling conditions	No	No—but see above
**Leaf sugar content**			
Tolerant (S68911) and sensitive (S160) inbred lines. 14/12 °C for 28 h at third leaf stage (Bilska-Kos *et al.*, 2016)	Yes—decreased phloem loading in sensitive line was observed; this leads to increased leaf sugars (not measured)	No	Yes—expression of genes involved in phloem loading
**Leaf expansion**			
Huang *et al.* (2013)	Yes	Yes—SNPs for shoot length identified	Yes—13 genes involved in biosynthesis, metabolism, cell division, and growth

Synthesis of studies including more than one genotype and measuring physiological responses to chilling stress. Studies are grouped according to the order of responses presented in Fig. 2. When describing each study, only chilling temperatures are included; control temperatures are omitted for brevity. Studies are listed under each applicable category but only described at the first instance.

^*a*^ QTL for a range of traits explained between 37% and 54% of the phenotypic variance in this study.

^*b*^ QTL explained up to 20% of phenotypic variance in this study.

^*c*^ Of these 18 genes, 10 were supported by other studies and three were novel.

^*d*^ These two QTL explained 19% and 6% of phenotypic variance.

^*e*^ The QTL on chromosome 6, probably at the end of bin 6.03, is located near to—and may be the same as—the QTL at bin 6.04 in the IBM2 2005 Neighbors 6 map, identified by Fracheboud *et al.* (2004). These may correspond to the *luteus11* locus which affects leaf colour (Rodríguez *et al.*, 2008).

^*f*^ A QTL related to leaf greenness on chromosome 3 was identified as being the same as a previously identified QTL related to photosynthesis, in a population derived from the same parent lines (Fracheboud *et al.*, 2004). Of the four QTL in late-sown plants, three were common with the early-sown plants.

^*g*^ This QTL explains 14% of phenotypic variation in chilling-induced albinism.

Studies measuring multiple traits across chilling-tolerant and chilling-sensitive genotypes frequently report relationships between physiological traits of interest. These relationships provide information about whether certain responses might indicate photoprotection or photodamage. For example, CO_2_ assimilation rate, Φ_PSII_, and chlorophyll content were all much lower in a chilling-sensitive line than in a chilling-tolerant line under prolonged chilling stress in a study by Fracheboud *et al.* (2004), indicating that reductions in Φ_PSII_ and chlorophyll content are more likely to be related to photodamage rather than protection. Similarly, in a study of two genotypes, there was a greater decrease in Φ_PSII_ under chilling stress in the chilling- sensitive line compared with the chilling-tolerant line (Sobkowiak *et al.*, 2014). Similar relationships were found between CO_2_ assimilation rate, Φ_PSII_, and chlorophyll content and chilling tolerance across a diverse collection of inbred lines (Lee *et al.*, 2002), again suggesting that decreased Φ_PSII_ is related to photodamage rather than being a photoprotective response. Additionally, an examination of 19 lines characterized for high or low Φ_PSII_ under chilling stress showed that the ‘high Φ_PSII_’ lines had a high CO_2_ assimilation rate, Φ_PSII_, and chlorophyll content under chilling stress (Hund *et al.*, 2005). Similarly, QTL linked to higher Φ_PSII_ under chilling stress derived from a mapping population originated from the chilling-tolerant parent (Jompuk *et al.*, 2005); and in a phenotypic screen for the effects of long-term chilling, a ‘favourable’ allele was linked to higher Φ_PSII_ (Fracheboud *et al.*, 2002). Taken together, these results indicate strongly that rather than lowering Φ_PSII_ for photoprotection, the maintenance of Φ_PSII_ is an important aspect of photosynthetic resilience to chilling stress. Similarly, most of the ‘favourable’ alleles at the QTL linked to a relatively smaller decrease in chlorophyll content under chilling stress were derived from the chilling-tolerant parent (Jompuk *et al.*, 2005). This indicates that in addition to limiting chilling-induced decreases in Φ_PSII_, the maintenance of chlorophyll is also advantageous during chilling stress, and suggests that the observed reduction in chlorophyll content may largely reflect photodamage rather than photoprotection.

Whilst the evidence provided by these studies supports the hypothesis that reduced Φ_PSII_ and chlorophyll content are linked to photodamage, repeated measurements made during a prolonged chilling stress also reveal a protective response that may occur as a result of priming. Fracheboud *et al.* (2002) showed that following an initial decrease in Φ_PSII_ in leaf 1 in response to chilling stress, which is likely to be a result of photodamage, Φ_PSII_ in leaf 3, that was subsequently developed under chilling stress, was also decreased. In this case, the down-regulation of Φ_PSII_ may indeed be part of photoprotective acclimatory responses. Interestingly, priming at a cool temperature prior to the imposition of a more severe chilling stress of 8 °C led to a less pronounced reduction in Φ_PSII_ at 8 °C, compared with plants that had been exposed directly to the 8 °C treatment with no priming (Sobkowiak *et al.*, 2016). This priming was more beneficial in the chilling-tolerant line than in the two chilling-sensitive lines used in the study where the sensitive lines always showed a greater reduction in Φ_PSII_ than the tolerant line.

Regarding the expression of photosynthetic genes, Li *et al.* (2019) examined transcriptional changes in a chilling-tolerant and a chilling-sensitive maize line. They found that the number of DEGs was much greater in the tolerant line during the first 24 h of chilling stress, with 1665 DEGs after 4 h and 3970 DEGs after 24 h; in the sensitive line there were 547 DEGs after 4 h and 1766 DEGs after 24 h. This may indicate either a more wide-ranging, or a more rapid, response in the tolerant line, although a more prolonged time course would be required to confirm this. Photosynthesis-related genes showed a faster response to chilling stress in the tolerant line, whilst genes related to the light-harvesting complexes decreased after 4 h in both lines, indicating an early photoprotective response. Interestingly, genes related to Φ_PSII_ were down-regulated after 24 h of chilling stress in the chilling-sensitive line only, which suggests that the tolerant line was not dependent on a photoprotective down-regulation of Φ_PSII_. Indeed, in the sensitive line, a greater decrease in *F*_v_/*F*_m_ coupled with an increase in *F*_o_ (the minimum fluorescence value measured after dark adaptation) indicated that photoinhibition and photodamage had occurred.

Many studies examining changes in chlorophyll content in response to chilling have identified both QTL and candidate genes, whilst few studies have identified candidate genes relating to the chilling-induced decrease in net CO_2_ assimilation rate (Table 1). CO_2_ assimilation is a complex trait, relying upon the amount, activation state, and activity of a range of enzymes, as well as the physiological status of the leaf, such as the status of the photosystems involved in the light reactions, plasmodesmatal conductivity to facilitate metabolite transfer, phloem loading rate, and—although only to a certain extent in C_4_ species—stomatal aperture. In contrast, chlorophyll content depends primarily on the synthesis and breakdown of chlorophyll, although of course the efficacy of chlorophyll in photosynthesis further depends upon its binding and coordination within the light-harvesting complexes. Because the regulation of chlorophyll content is less complex than the regulation of photosynthesis, it may be more straightforward to use chlorophyll content for the identification of candidate genes to enhance chilling tolerance, rather than using CO_2_ assimilation or Φ_PSII_. Candidate genes involved in the regulation of chlorophyll content under chilling stress have been identified, with more studies reporting candidate genes for chlorophyll than any of the other traits, with the exception of gene expression changes under chilling stress (Table 1). In spite of the relative paucity of candidate genes related to net CO_2_ assimilation or to Φ_PSII_ under chilling conditions, the fact that many studies have identified QTL (or SNPs) for these two traits suggests that it will be possible to establish some candidate genes in the near future. The relative contribution of these QTL to the level of each trait in response to chilling and the persistence of this contribution in different genomic backgrounds and across different environments will be important determinants of their utility in breeding programmes.

Although several of the photosynthetic and photoprotective responses to chilling have already been used for genetic mapping studies, this is not the case for variation in antioxidant capacity in response to chilling. Candidate genes involved in antioxidant capacity were identified both as a result of mapping variation in chilling tolerance indices (Huang *et al.*, 2013) and by making transcriptomic comparisons between tolerant and sensitive lines (Sobkowiak *et al.*, 2014; Jończyk *et al.*, 2021), but their involvement awaits further experimental verification since antioxidant capacity was not directly measured in any of these studies. Following up this work with direct measurements of antioxidant capacity, or with genetic mapping of variation in antioxidant capacity in response to chilling may provide another piece of the puzzle as we move towards a more complete understanding of chilling-tolerant photosynthesis in maize. Interestingly, the accumulation of zeaxanthin was negatively correlated with chilling tolerance in a study of maize genotypes differing in chilling tolerance (Fracheboud *et al.*, 2002). While the accumulation of zeaxanthin is associated with a sustained form of NPQ (*q*_Z_; Nilkens *et al.*, 2010), it is also a potent ROS scavenger (Havaux *et al.*, 2007), which leaves two possible explanations for the observed negative relationship. On the one hand, the impact of zeaxanthin on NPQ may depress maize photosynthetic efficiency in response to chilling, as suggested by Fryer *et al.* (1995). Alternatively, the increased accumulation of zeaxanthin in sensitive genotypes could reflect a greater need for photoprotection in these genotypes. The fact that lower Φ_PSII_ and CO_2_ assimilation across the sensitive genotypes in Fracheboud *et al.* (2002) also correlated strongly with proxies for larger light-harvesting antennae, which would increase excitation pressure per PSII reaction centre, would seem most consistent with the second explanation.

Overall, many QTL relating to the physiological components of photosynthetic chilling tolerance in maize have been identified, particularly with respect to CO_2_ assimilation, Φ_PSII_, and chlorophyll content. However, it is striking that relatively few candidate genes have been identified when considering the broad range of studies examined in this review (Table 1). This may be due to the fact that many traits are polygenic, meaning that whilst QTL may be readily identified, pinpointing genes of interest that are responsible for the traits in question is altogether more difficult. For example, CO_2_ assimilation is an emergent property that depends upon a plethora of physiological and molecular players, meaning that a wealth of genes underpins this complex trait. Likewise, candidate genes for Φ_PSII_ are relatively rare and no candidate genes for NPQ have been identified (Table 1). Whilst transcriptomic analysis of photosynthetic gene expression by definition identifies the expression of photosynthesis-related genes, even chlorophyll content—which is a comparatively simple trait related to chilling tolerance of photosynthesis—does not have many associated candidate genes in the studies reviewed here, whilst for leaf sugar content the candidate genes that have been identified are involved in phloem loading rather than being more directly involved in sugar metabolism (Table 1). Finally, some genes relating to antioxidant activity and to leaf expansion have been identified, but none for ABA with respect to chilling tolerance (Table 1). It should also be noted that QTL mapping is much easier than the definitive identification of candidate genes and, since the draft genome of maize was published relatively recently (Schnable *et al.*, 2009), the possibility of identifying candidate genes is rather new in maize compared with model species such as *Arabidopsis*. Furthermore, many of the studies reviewed here focused on meeting breeding objectives, for which QTL are instrumental but the identification of specific candidate genes is generally not necessary. From a physiological perspective, elucidating the causal sequence for a trait increases the possibility of successfully understanding the underlying mechanism, so studies focused on physiological goals may be more likely to pursue the identification of candidate genes rather than QTL.

Looking to the future, there exists significant diversity in Φ_PSII_ between breeding groups and populations (Strigens *et al.*, 2013), and this could be exploited for the development of chilling-tolerant germplasm. Future studies might investigate the genetic basis of variation in the other physiological traits we have highlighted in this review, and the contribution of this variation to chilling tolerance or susceptibility. The identification of more candidate genes will also be important, as outlined above. Due to the complexity of several responses with respect to photoprotection and damage, the use of experimental time courses in combination with phenotyping across the broader spectrum of physiological responses to chilling as outlined here will be critical for appropriate interpretation and may lead to the identification of more stable QTL and candidate genes.

## High-throughput breeding approaches

Having examined the physiological basis for photosynthetic chilling tolerance and the genetic variation for this tolerance revealed in a range of populations and responses, we now return to our central question: Can we improve the chilling tolerance of maize photosynthesis through breeding? Whereas most of the responses to chilling appear to show intraspecific genetic variation in maize, appropriate interpretation of this variation requires determination of several responses in parallel across large populations.

### Physiological breeding for improving photosynthetic chilling tolerance

Physiological breeding aims to incorporate physiological trait measurements into breeding programmes (Reynolds and Langridge, 2016). Such measurements can be more time-consuming and labour-intensive, but are valuable for understanding the physiological responses of plants to different stresses, especially when combined with powerful QTL analysis in the breeding context. High-throughput approaches for measuring physiological traits are therefore of great benefit; two such approaches are chlorophyll fluorescence, which has been discussed above, and reflectance spectroscopy. While measurements of Φ_PSII_ using chlorophyll fluorescence may be readily applied in a high-throughput manner (Hund *et al.*, 2005) and can be tailored to specific traits of interest (Maxwell and Johnson, 2000; Baker, 2008; Murchie and Lawson, 2013), several additional techniques to cover more of the nine key responses to chilling in parallel are now available. In particular, reflectance spectroscopy offers another high-throughput approach. A major advantage of this technique is that similar to fluorescence techniques, a rapid measurement (~1 s) enables the simultaneous estimation of a suite of metabolic and physiological parameters of interest via correlative models (Yendrek *et al.*, 2017; Ely *et al.*, 2019; Burnett *et al*., 2021*a*, *b*, *c*). For example, following the development of training datasets and models which are appropriate for the genotypes and traits of interest, the maximum carboxylation rate of Rubisco (Serbin *et al.*, 2012; Meacham-Hensold *et al.*, 2020), leaf protein and sugar content (Ely *et al.*, 2019), ABA (Burnett *et al.*, 2021*b*), and chlorophyll content (Yendrek *et al.*, 2017) may all be predicted from a single hyperspectral measurement. Taken together, these parameters provide a more holistic picture of the physiological response to chilling stress and would enable quantification of photoprotective mechanisms as well as foliar damage caused by chilling. Chilling tolerance can trade off against other useful desired traits in maize (Frascaroli and Revilla, 2019); this furthers the requirement for a holistic perspective when breeding for chilling tolerance.

Hyperspectral reflectance measurements are rapid and, once equipment has been purchased, the costs per measurement are negligible. Many options are available, including leaf clips for leaf-level measurements and unmanned aerial vehicle (UAV) platforms for screening fields at the plot level. Currently, hyperspectral measurements typically need calibration within each system of interest before they can be used for trait identification. However, it is possible to predict the structural trait leaf mass per unit area (LMA) using reflectance data alone (Serbin *et al.*, 2019), and in the future it will become increasingly feasible to predict traits of interest based on generalized models once models have been trained on wider-ranging datasets and the leaf structural and optical properties have been accounted for. This will significantly augment the utility of hyperspectral reflectance for breeding programmes.

A physiological breeding approach will be instrumental when dealing with multiple complex stresses. Rarely does a single stress occur. Rather, the dynamic field environment can impose stresses in combination, such as heat and drought stress during hot summers, or chilling and high light stress in temperate spring seasons; considering biotic stresses such as pathogens adds a further dimension. Interestingly, plant responses to stresses often overlap or compound each other. For example, a population of 233 maize recombinant inbred lines (RILs) derived from a drought-tolerant and a drought-sensitive parent was subsequently shown to contain a large degree of segregation in chilling tolerance, demonstrating strong overlap between chilling and drought stress tolerance (Fracheboud *et al.*, 2002). Levels of ABA and proline, which are involved in responses to and alleviation of drought stress, have also been shown to be involved in acclimation to chilling stress in maize (Dory *et al.*, 1990; Xin and Li, 1993; Revilla *et al.*, 2005). Chilling temperature stress generates a distinct metabolic and molecular fingerprint, but also leads to responses that are shared with other stresses (Geange *et al.*, 2021). Understanding the hallmark signs of enhanced tolerance to a combination of stresses is essential for breeding maize for an increasingly chaotic and unpredictable future climate.

### Breeding for enhanced chilling tolerance must consider crop phenology and target environment

The goal of a breeding programme must be carefully considered when designing experiments destined to inform the selection and development of maize germplasm. Both field and controlled environments have limitations when it comes to conducting chilling stress experiments; combining both approaches, with multiple years and locations, is recommended for understanding and exploiting the true variation in maize chilling tolerance (Frascaroli and Revilla, 2019). The timing of the chilling stress is also important. Breeding chilling-tolerant maize able to withstand long-term chilling temperatures and acclimate to chilling conditions may give a different outcome than breeding maize able to withstand short-term ‘cold snaps’ in otherwise mild conditions. Cold snaps at any stage of growth can impact yield—by reducing germination, slowing vegetative growth and development, or inhibiting reproductive processes. Chilling tolerance does not always increase yield, and indeed there can be a trade-off between yield and stress tolerance (Revilla *et al.*, 2005), although historic maize yield improvement has been shown to be strongly related to enhanced stress tolerance (Tollenaar and Wu, 1999). Successful breeding for chilling tolerance must consider which growth stage is of particular interest and determine which trait or combination of traits to target. Improvements in resource use efficiency are often only revealed when plants are in stressful conditions (Tollenaar and Wu, 1999). In this context, we note that chilling stress at the reproductive stage in maize is relatively understudied, and may be an important area for further research in an increasingly erratic climate.

### Transgenic approaches for improving chilling tolerance of photosynthesis

While this review focuses on pre-existing variation in chilling tolerance of photosynthesis in maize, and the genomic regions related to this tolerance which may be utilized in breeding programmes, it is worth noting that genetic modification approaches also offer valuable tools for improving photosynthesis and chilling tolerance. For example, increasing Rubisco and electron transport capacity can improve the photosynthetic performance of C_4_ plants; Rubisco is predicted to have a greater effect on chilling recovery than other photosynthetic enzymes in the C_4_ pathway (Sales *et al.*, 2021). The overexpression of Rubisco large and small subunits, in concert with Rubisco Assembly Factor 1 (RAF1), increased maize Rubisco content by >30% (although Rubisco activase is probably a vital factor for translating this increased enzyme content into a proportional increase in photosynthetic activity); this overexpression of Rubisco can speed recovery following chilling stress (Salesse-Smith *et al.*, 2018). Transgenic introduction of chilling-tolerant PPDK into maize lowered the threshold for chilling stress in the extracted enzyme and increased photosynthesis by 23% under chilling conditions of 8 °C (Ohta *et al*., 2004, 2006) whilst introducing the osmoprotectant molecule glycinebetaine transgenically into maize increased photosynthesis and reduced chilling damage (Quan *et al.*, 2004).

Transgenic work carried out in other species demonstrates useful proofs of concept, although we acknowledge that a detailed discussion of this topic is outside the scope of the present review. For example, the *AlSAP* gene from the grass *Aeluropus littoralis* has been successfully expressed in rice where it increased photosynthesis and stress tolerance when plants were exposed to a chilling treatment as well as other abiotic stresses (Ben Saad *et al.*, 2012). Work in *Arabidopsis* has shown that the CBF/DREB1 transcription factors are important for the chilling response (Miura and Furumoto, 2013), and transgenic CBF/DREB1 transcription factors from *Arabidopsis* have been used to improve chilling tolerance in tobacco and wheat (Sanghera *et al.*, 2011). Multiple genes, including genes from the *CBF/DREB1* family, have been transgenically introduced into rice to increase chilling tolerance, highlighting the complex nature of chilling tolerance and its regulation (da Cruz *et al.*, 2013).

Finally, the activation of latent genes already present within the genome, and a greater understanding of genetic regulatory mechanisms, are important elements of increasing chilling tolerance (Revilla *et al.*, 2005). Transgenic approaches may also be used to investigate the presence and function of genes that already exist within the species of interest. For example, a study overexpressing a stress-responsive binding factor from the Antarctic grass *Deschampsia antarctica* in rice used RNA-seq to identify a candidate set of genes involved in the rice chilling stress response, putatively regulated by the *D. antarctica* binding factor (Byun *et al.*, 2018). Finally, gene editing using CRISPR/Cas9 can be used to introduce specific beneficial alleles into germplasm (Waqas *et al.*, 2021).

### Expanding allelic diversity for chilling tolerance

Considering conventional breeding methods, broad genetic diversity is important for breeding (Revilla *et al.*, 2005), and this includes diversity encompassing pre-existing variation in photosynthesis (Faralli and Lawson, 2020). Introducing germplasm from varieties or wild crop relatives adapted to high altitude and/or low temperature can aid chilling tolerance of crops (Sanghera and Wani, 2008). In maize, the use of germplasm from different environments of origin is a useful means of increasing allelic diversity for improving chilling tolerance. For example, it was shown that many Mexican highland maize landraces contain several introgressions obtained from a highland subspecies of the wild relative teosinte (*Zea mays* ssp. *mexicana*). One of these introgressions, a large chromosome inversion segment, could indeed be linked to increased chilling tolerance and improved photosynthesis under chilling conditions, including increased Φ_PSII_ and increased chlorophyll gene expression (Crow *et al.*, 2020). The use of maize lines developed in temperate regions may also improve chilling tolerance. In a study comparing 598 European inbred lines, several ‘favourable’ alleles for Φ_PSII_ were identified, especially across the European flint lines (Revilla *et al.*, 2016). Local landraces may be used to introduce additional diversity into elite germplasm, but due to their heterozygous nature these are more difficult to use directly for breeding. Recent efforts to create doubled-haploid lines produced from landraces therefore provide a useful resource for understanding and exploiting the genetic and phenotypic diversity available in maize landraces (Hölker *et al.*, 2019).

Finally, it will be important to integrate agronomic and genetic approaches to achieve future food security (McKersie, 2015). Besides breeding for increased resilience, agronomic techniques can be employed to increase chilling tolerance. For example, the application of ‘climate-smart agriculture’ regimes such as altered planting times, the application of exogenous plant growth regulators, and seed coating and seed priming can further help to mitigate the effects of low temperatures (Waqas *et al.*, 2021). Just as priming with a moderate chilling stress can alleviate a severe temperature stress in maize plants (Capell and Dörffling, 1993; Sobkowiak *et al.*, 2016), seed priming has been shown to improve antioxidant levels and growth under chilling stress (Li *et al.*, 2017).

## Conclusion

Whilst the relationship between photosynthesis and yield is complex, photosynthesis is a major contributing factor to yield (Sarquís *et al.*, 1998; Simkin *et al.*, 2019) and the chilling tolerance of photosynthesis is an important component of improved performance of maize under chilling temperatures (Dwyer and Tollenaar, 1989; Tollenaar and Wu, 1999). Here we have identified nine traits that are pivotal in the maize chilling response: carbon assimilation; electron transport; the expression of photosynthetic genes; NPQ; chlorophyll content; ROS; ABA; leaf sugar content; and leaf expansion. Since the chilling tolerance of photosynthesis is a complex breeding goal with multiple phenotypic and genotypic components, we advocate for a multi-trait holistic approach that takes specific phenological and geographical considerations into account for successful breeding for chilling tolerance of photosynthesis. Breeding for increased chilling tolerance of photosynthesis by exploiting the substantial natural genetic variation for traits aligned with key chilling responses will improve maize yields in cooler climes and contribute to meeting the significant global food security challenges faced by humankind.
